# An empirically-derived approach for investigating Health Information Technology: the Elementally Entangled Organisational Communication (EEOC) framework

**DOI:** 10.1186/1472-6947-12-68

**Published:** 2012-07-12

**Authors:** Andrew Georgiou, Johanna I Westbrook, Jeffrey Braithwaite

**Affiliations:** 1 Centre for Health Systems and Safety Research, Australian Institute of Health Innovation, Faculty of Medicine, University of New South Wales, Sydney, Australia, 2052; 2 Centre for Clinical Governance Research in Health, Australian Institute of Health Innovation, Faculty of Medicine, University of New South Wales, Sydney, Australia, 2052

## Abstract

**Background:**

The purpose of this paper is to illustrate the Elementally Entangled Organisational Communication (EEOC) framework by drawing on a set of three case studies which assessed the impact of new Health Information Technology (HIT) on a pathology service. The EEOC framework was empirically developed as a tool to tackle organisational communication challenges in the implementation and evaluation of health information systems.

**Methods:**

The framework was synthesised from multiple research studies undertaken across a major metropolitan hospital pathology service during the period 2005 to 2008. These studies evaluated the impact of new HIT systems in pathology departments (Laboratory Information System) and an Emergency Department (Computerised Provider Order Entry) located in Sydney, Australia.

**Results:**

Key dimensions of EEOC are illustrated by the following case studies: 1) the communication infrastructure between the Blood Bank and the ward for the coordination and distribution of blood products; 2) the organisational environment in the Clinical Chemistry and Haematology departments and their attempts to organise, plan and control the processing of laboratory specimens; and 3) the temporal make up of the organisation as revealed in changes to the way the Central Specimen Reception allocated, sequenced and synchronised work tasks.

**Conclusions:**

The case studies not only highlight the pre-existing communication architecture within the organisation but also the *constitutive* role communication plays in the way organisations go about addressing their requirements. HIT implementation involves a mutual transformation of the organisation and the technology. This is a vital consideration because of the dangers associated with poor organisational planning and implementation of HIT, and the potential for unintended adverse consequences, workarounds and risks to the quality and safety of patient care. The EEOC framework aims to account for the complex range of contextual factors and triggers that play a role in the success or otherwise of new HITs, and in the realisation of their innovation potential.

## Background

### Introduction

Evaluation can be defined as the systematic determination of the quality, value or importance of, for example, programs, projects or institutions [1]. Although evaluations are generally conducted to identify areas for improvement or provide an overall assessment, they (like any evaluand) are susceptible to failure [2]. Evaluations may ask the wrong question, adopt an inappropriate method or paradigm [3] or even just fail to notice “the elephant in the living room.” [4] There is no perfect or singular evaluation approach. Evaluation of health information technology (HIT) is particularly challenging. HIT is inherently disruptive and has the capacity to transform the organisational landscape and impact on professional responsibilities and roles [5]. Systematic reviews of HIT studies continue to highlight the complex, variable and fragmented nature of evidence in this field [6-9].

The HIT challenge underscores the importance of employing theory-based approaches that can help to integrate and highlight the significance of findings and improve our understanding of how and why things happen [10-13]. As an example of this, consider the results of studies suggesting that a Computerised Provider Order Entry (CPOE) system has attained its goal and is deemed to be working successfully [14-16]. Such findings are valuable for many purposes, but they may not necessarily explain what it is about the system within its defined context that made it work. In order to appreciate the innovative potential of HIT more fully we need to look beyond its technological features and incorporate the organisational communication structures and social-material setting within which the technology is embedded and enmeshed [17]. In the end, it is not programs (or technologies) in and of themselves that work, but the resources they offer to enable people to make them work within a complex, adaptive environment exhibiting multi-faceted cultural features [12]. Evaluating what works, for whom and in what circumstances, as Pawson and Tilley [18] suggest, can help fill in the broader research jigsaw puzzle that can contribute to an understanding of the HIT and the role it can play as part of an organisation’s “innovation journey” [19,20].

### Organisational communication and health informatics

While Pawson and Tilley [18] encourage a focus on context, mechanisms and outcome (CMO) we wish also to highlight the role of approaches that consider the constitutive function that communication plays in organisational processes [21,22]. Early organisational communication studies, prominent in the US during the mid-part of the last century, emphasised the importance of channels of information which incorporated clear lines of accountability and responsibility and their role in ensuring effectiveness, efficiency and output [23-25]. More recent scholarship has presented organisations as highly complex, adaptive and emergent organisms where communication is seen more as a *multi-transactional* process incorporating reverberating feedback and iterative confirmation involving two or more people within a multi-faceted environment [22]. From this perspective, communication processes can be seen as part of the *social glue* facilitating organisational functioning [26,27]. These processes are *elemental* because they undergird the way that organisations operate, but also deeply *entangled* as interrelated components of the way that organisations make sense of their environment, coordinate their activities and make decisions about their future. In essence, communication processes need to be studied because they are the sociological and organisational DNA that make things work [28]. We label initiatives which attempt to investigate this phenomenon the *Elementally Entangled Organisation Communication (EEOC)* approach.

Organisational communication approaches in the way described have yet to be widely utilised by the health informatics community, at least explicitly [29]. Giuse and Kuhn’s outline of the challenges identified by the Heidelberg Health Information Systems Working Group conference in 2002 drew attention to an apparent disregard for communication among clinical users [30]. Moreover, as Kuziemsky et al. highlight, existing research often fails to consider the role of communication in the context of specific team structures, processes and outcomes [31]. Communication failures, problems or misalignments are widely seen to be a central reason for poor quality health care today, but understanding the dynamics of these failures and their complex connection with hierarchy, social roles and organisational structures are not so well understood [32]. The obvious implication of this is the need for theoretical approaches which can be employed to deepen our understanding of what is happening and why it is happening [11].

Previous research approaches have tended to describe organisations as fixed entities or containers through which information is transmitted and communicated to internal and external audiences [33]. However, as per Weick, organisations are more than this: they are dynamic entities comprising people enmeshed in the processes of sense making, organising and interpreting their environment [34,35]. Communication process are therefore an essential part of the process of establishing and maintaining the ongoing, interconnected behaviours that contribute to the makeup of an organisation [33]. This is particularly relevant for research involving health information systems which have a *disruptive* ability to change the role communication plays in organisationally linking people and activities across space and time [35]. In this way we believe that organisational communications perspectives can complement, underpin and build on some of the better known approaches such as socio-technical [36,37], workflow [38,39] and system approaches [40-42].

EEOC draws on rich sources of organisational communication scholarship which have been iteratively assessed and applied to empirical data to establish a novel innovative theoretical tool to inform future research. There are compelling reasons for the development of EEOC as a theoretical lens for HIT research. Firstly, health care itself is essentially an embedded, collaboratively-oriented set of organisational activities which rely on communication within and between groups to coordinate care [29,43]. Poor coordination of care is often cited as one of the main causes of inadequate services and adverse patient events [44,45]. Secondly, new technologies disrupt communication activities and influence the organisational structure and process [46]. This can involve role changes [47], transformations in the way that departments interact with each other [43,48,49], altered cultural constraints and enablers [50], adaption to changing social networks [51-53] and modified infrastructure configurations [54]. Thirdly, EEOC implicitly requires attention to the socio-material and temporal-spatial requirements of HIT implementation particularly as regards how and where work is allocated, coordinated, enacted and synchronised [55]. This is because new technologies can affect the way that clinical work is carried out, the speed with which it is undertaken and even the setting (e.g., point of care or tele-health) in which it is performed [56].

### Objective

The aim of this paper is to present an EEOC framework as a theoretical lens that can be applied to the study and implementation of HIT systems and as a tool for understanding and unlocking their innovation potential. The framework was synthesised and developed from an extensive body of empirical research over a period of three years that investigated the impact of a new Laboratory Information System (LIS) and CPOE system on a large metropolitan pathology service in Sydney, Australia.

## Methods

### Research setting

The research was conducted in a pathology service employing over 300 staff and a 66-bed Emergency Department (ED), both located at a major metropolitan tertiary referral hospital in Sydney, Australia. The pathology service is responsible for seven major hospitals covering an estimated population of 1.33 million. The study was carried out across five pathology departments (Clinical Chemistry, Haematology, Central Specimen Reception, Microbiology and the Blood Bank) during the period August 2005 to August 2008. In November 2005 these departments had their previous LIS replaced by the Cerner Corporation’s (Kansas City, USA) Pathnet system which automates clinical and managerial pathology data processes. In January 2006 this system was integrated into a new hospital-wide CPOE system called PowerChart (version 2004.01) which replaced hand-written paper requests for pathology and other services. Ethics approval to study the HIT implementation (Project No. 2005/058 and Project No. 2007/077), was provided by the relevant Health Service Research Ethics (HREC) committees.

### Data collection

The research study conducted 16 focus groups (involving 68 participants) and 141 interviews (75 participants) and 43 hours of observation. All participants provided their informed consent. Six of the focus groups, seven interviews and four hours of observation were undertaken in the Emergency Department. In total, this involved nine ED physicians and 20 registered nurses. The focus group and interview sessions were transcribed and resulted in 531 single spaced A4 pages of text. A researcher’s log collected notes from all sessions along with memos and notes on the investigation process amounting to 203 entries and a total of 243 A4 pages of text. The research log provided a flexible tool for reviewing and reflecting on the progress of the research [57]. Theoretical sampling served as the guiding focus for the selection of cases (e.g., pathology departments and ED setting) and study participants.

### Analysis

Field materials were analysed in order to relate data and concepts, building a viable real world narrative, synthesising participants’ behaviours, attitudes and discourse, and mapped to their situation [58,59]. This was achieved by a team of research experts in areas of qualitative data analysis and involved a process of constant comparison of data for similarities and differences [60]. NVivo software was used by AG to undertake an initial open coding of all interview and focus group transcriptions [61]. Axial coding (involving the whole research team) was performed whereby initial codes, indicators and concepts were exposed to more and more data, and then elaborated on, and transformed into robust categories leading to more refined analytical levels relevant to the topic under investigation [62].

### Theory development

This work initially adopted a realist approach to achieve its research aim, drawing on Pawson and Tilley’s context-mechanism-outcome framework [18]. Realist researchers acknowledge the existence of the observed world but remain mindful that our understanding of that world is theory-laden, socially constructed and fallible [63]. Since the real world is differentiated, stratified and made up of an assortment of interacting, emergent events, objects, materials and behaviours, it follows that robust knowledge should be a product of multiple methods, triangulated across a range of perspectives [19]. Different research methodologies and strategies are more appropriate for some purposes than others. A combination of approaches can facilitate a more comprehensive investigation [37,64]. In this study the realistic evaluation was broadened with a multi-method triangulated approach that incorporated an organisational communication perspective. This allowed for the EEOC framework to emerge orthogonally from the data. The framework brought together, helped focus and provided meaning to multiple components of the study. Rather than the end-product of the study the framework was iteratively refined and developed in the course of the research, serving as a means to design questions, guide the selection and analysis of data and to help formulate explanations about events and trends [65].

## Results

### Organisational communication as an orienting framework

**Figure 1:**
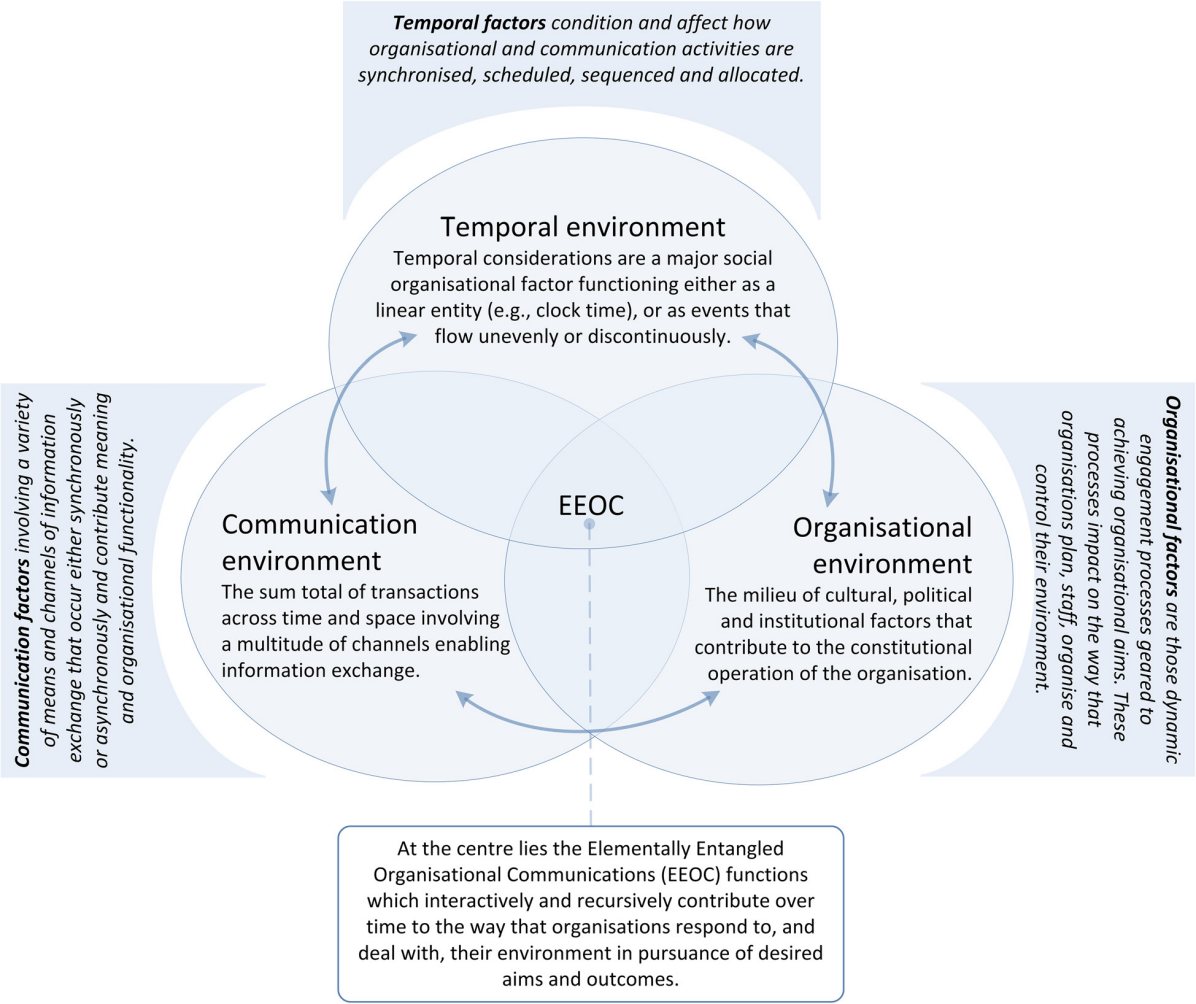
A conceptual depiction of the components and interconnections of the EEOC framework.

The introduction of HIT can intrude on the way that information is processed, decisions are made, cultures are formed and upheld, and how the organisation is controlled. We draw on a set of three case studies to illustrate the impact of the new LIS and CPOE system on different organisational communication dimensions of the pathology service and its relationship to other parts of the hospital. Figure 1 depicts the key dimensions that make up the EEOC framework [66], emphasising the relationship that the organisational infrastructure has with management functions, communication networks and the temporal activity of the organisation. These components of the framework highlight a number of guiding themes which are then used to interrogate the findings and orient the analysis and understanding of the results. For instance, some of the guiding themes for the communication environment may include considerations about what type of information is exchanged and how it is communicated and processed. Alternatively, key themes for the temporal environment may consider the role of information and communication in the allocation of work, the time taken for it to be carried out and how it is allocated. For the organisational environment, the key considerations may turn on how work is planned, organised, staffed or coordinated.

### Case study I -The Blood Bank’s communication environment

The pathology test procedure can broadly be described as a process that begins with a doctor making a request for a test on a specimen, which is then transported to the laboratory for analysis and ends with a test result that is reported back to the doctor. Communication across the laboratory–ward interface can take on various forms, either as synchronous exchange (e.g., at the same time using a telephone) or asynchronous exchange (e.g., message or note). The seemingly linear process of *test order, analysis and result*, is accompanied by a complex array of actions which forms part of a collaborative laboratory–ward effort involving many different groups [67] and reliant on a number of associated processes. In this sense communication is both *elemental* to the purpose of the laboratory, and *entangled* within different sectors and parts of the laboratory and wider hospital environment.

The role of the Blood Bank in the hospital pathology service is to provide compatible blood components for patients. It is also responsible for undertaking its own range of tests which are concerned with blood grouping, antibody screening and identification and pre-transfusion testing. In contrast to other pathology departments, the Blood Bank’s role involves more than just providing a test result, but includes the dispensation of a blood product [68]. This component of the Blood Bank’s role exerts an important influence on the department’s communication environment. Hence, the Blood Bank process starts with a prescription for a blood product from a physician. This is communicated to the Blood Bank by an official request form, telephone call or fax. The product is then prepared and stored awaiting notification that it needs to be delivered. Both the Blood Bank and clinical staff have a shared responsibility to ensure the correct communication of patient details and labelling of specimens to avoid patient identification error. Even after the delivery of a blood product the Blood Bank needs to be notified that it has been received. Red cell products cannot be left out for more than 30 minutes. To guard against this occurrence the Blood Bank rings the ward to confirm that the responsible physician is aware of the blood product arrival.

The communication transaction described above relies on high levels of synchronisation, accuracy and collaboration with the ward as part of a two-way process of message reinforcement [56,69,70]. This is vital to conveying accurate meaning [27] and ensuring the delivery of safe patient care [68]. Prior to the introduction of CPOE, the method that hospital staff used to communicate orders to the Blood Bank was predominantly synchronous, using the telephone or the fax machine which triggered an alarm system to notify Blood Bank staff of the presence of a request. The switch from a synchronous exchange to an asynchronous one (which involved posting a message on the system) implied a major change in the collaborative relationship between the hospital ward and the Blood Bank and was a cause of some concern. This is because of the fear that the department may fail to notice or not be adequately notified of the existence of an electronic request which may sit unnoticed in system. This trepidation led to the indefinite postponement of electronic requests for blood products.

This communication factor has major implications for the implementation of CPOE systems which incorporate many asynchronous modes of communication. On the one hand, the new CPOE system greatly improved the monitoring process within the department by providing an audit trail of each of the important steps in the Blood Bank process, which facilitated accountability within the department. On the other hand, the Blood Bank’s concern about the possibility of messages from the ward going unnoticed, caused considerable concern about the safety and adequacy of the system’s warning and notification mechanisms, to the point that two years after the introduction of CPOE it was still not used to order blood products [56].

### Case study II – the Clinical Chemistry and Haematology organisational environment

The management of every organisation can, instrumentally speaking, be said to incorporate the classic management functions of planning, organising, staffing and controlling [71]. Each of these tasks is connected to a communication dimension. In order to *plan* it is important to access information with which to forecast and predict the course of the future. The *organisation* of work requires people and resources to be set out within established communication channels. *Staffing* includes communication required for the management of resources and *controlling* involves the coordination of resources using the exchange of information. Changes in communication patterns can result in new ways of interacting that can alter the organisational environment and the way of doing things. It follows therefore that the success or otherwise of HIT is heavily dependent on its suitability and fit within the unique organisational communication setting in which it is deployed.

The Clinical Chemistry department is concerned with the analysis of blood and other body fluids for chemical components. The Haematology department is involved in the study of blood along with its cellular elements, and the diseases of the blood and blood forming tissues. Together these two departments are responsible for the great bulk of tests across the pathology service. This means they are reliant on a series of tasks such as the accessioning (assignment of a laboratory identification number) of new specimens, specimen preparation, sample distribution, test analysis and result verification. These tasks are intrinsically connected to the flow of information, and therefore to the Laboratory Information System (LIS) [72]. The pathology department LIS can be described as at the centre of most pathology laboratory functions including work flow management, specimen tracking, data entry and reporting, interfacing with other systems, archiving and inventory control – activities that are related to the way that the departments plan, organise, staff and ultimately control their environment [73]. Information and the capacity to receive, process, and communicate it in a timely and accurate manner are crucial organisational functions. This connection suggests that in order to understand how the pathology department responds to challenges, like the introduction of a new LIS/CPOE system, it is necessary to examine how laboratory information is obtained, processed, stored and transmitted [27].

Before the implementation of the new LIS/CPOE both Clinical Chemistry and Haematology operated middleware systems which added functionality to their existing LIS and helped facilitate result handling, tracking specimens and storage [73]. Clinical Chemistry utilised middleware for result interpretation, tracking and handling of test specimens. The department has to keep control of its specimens and aliquots (daughter tubes) and be able to locate them when needed. The existing homegrown system allowed the laboratory to identify the processes that a specimen had been through, and to ascertain what processes were still required. It also provided the laboratory with a designated position where the specimen was to be stored. The new system did not replicate this process, requiring the laboratory to manually allocate a rack and storage position.

For Haematology, middleware played an autoverification role which incorporated checks on reference ranges, quality control, critical values, delta checks, dilution needs, instrument flags and laboratory review policies [73]. The new Cerner Pathnet LIS did not replicate this role [74]. This situation required the laboratories to undertake a complex set of negotiations with the software vendor to compensate for these missing functionalities and to devise a system to replicate the tracking and monitoring functions of the previous middleware system. This led to the development and introduction of a new “Specimen Orderable Status” (SOS) program which read specimen barcodes and indicated whether results had been validated or not, identifying those results that needed to be manually validated [74].

The experiences of the Clinical Chemistry and Haematology departments highlight how each new technology needs to be implemented in the context of existing infrastructures and social practices [75]. It also shows how the pre-existing organisational and communication environment can affect the way that work is allocated, organised and controlled and how information is communicated.

### Case study III - Central Specimen Reception’s temporal environment

The way that time is scheduled, partitioned and organised plays a fundamental role in the life of an organisation [76]. It is hard to obtain meaning about the function of an organisation without due consideration to its temporal activities. As per Weir et al., all tasks related to laboratory testing can be considered to have a time element. This can include consideration about the time a task is due, the length of time it takes to complete a task or even the period that is required for each task. If this information is not known, e.g., when the patient was last seen, the number of times a test has been repeated, it usually means that people have to revert to verbal communication or other workaround to confirm information [70].

Pathology services are geared to ensuring timely laboratory test results (e.g., test turnaround times) [77,78]. This entails consideration of clock time which flows evenly and continuously, can be easily quantified and is freed from contingent events [79]. Many decision support features of CPOE systems are predicated on ensuring the appropriateness of test ordering through prompts to avoid the ordering of redundant tests, i.e., tests which have been reordered within an inappropriate time frame and will provide no additional information [80]. However, the temporal make up of a pathology service is made up of a lot more than just considerations about clock time. There are also workflows which are subject to periodic patterns or events which flow unevenly and discontinuously and are highly contingent on a number of factors including staff availability or hospital routines and practices [76].

In the case of the Central Specimen Reception area of the pathology service, blood collectors (phlebotomists) perform two rounds of specimen collections per day (8.00 am and 1.00 pm). Before the implementation of CPOE this involved the collectors visiting a ward to access hand-written forms containing laboratory test requests that were usually stored within a special ward location in a filing basket or box. In this process, the collectors were required to check the details of each request, match the hand-written request with each patient, identify any duplicates, find the correct patient and then proceed with the collection [66]. The blood collectors’ temporal cycle was thoroughly transformed after the introduction of CPOE. The sorting and collation process now occurred within the department using a printout of the required collections. Blood collectors were no longer required to identify and check for duplicate orders or provide hand-written labels to accompany specimens. The change in collectors’ patterns of work highlights the impact that technology can have on different temporal dimensions [81] of work including on the: a) *duration* or amount of time taken to complete activities (i.e., no longer necessary to sort out paper requests); b) *sequence*, or the order that activities are undertaken (i.e., patient-related information is now organised at the start of each shift); c) *location* of activities (i.e., the task of organising and collating blood collection requests shifted from the ward to the Central Specimen Reception area); d) the *periodic cycle* with which tasks are carried out along with alterations in the rhythm and intensity of the different areas of work; (i.e., a change in the time spent on a number of tasks); and finally, e) consequent change to *deadlines* and *expectations* associated with parts of the work cycle (i.e., changes in the time taken to complete tasks).

## Discussion

### Organisational communications as theoretical lever to advance innovation

The EEOC framework is premised on the understanding that technology should not be seen as separate from the other parts of an organisation, but deeply embedded in organisational communication processes. New technologies interact with the rest of the organisation and should therefore be viewed within this wider perspective [76]. Table 1 outlines key guiding themes from the EEOC framework alongside findings from each of the case studies. The case studies highlight not only the pre-existing communication infrastructures (e.g., the essential collaboration involved between the Blood Bank and the ward in coordinating safe and timely blood products to patients, particularly related to how information is processed) but also the way organisations go about addressing their requirements (e.g., the Clinical Chemistry and Haematology departments attempt to organise, synchronise and control the tracking and monitoring of specimens as part of how work is controlled). A realist analysis would consider these as part of the *contextual setting* of the pathology service. In order to comprehend the innovative capacity of new technology there needs to be an assessment of its impact on the temporal make up of the organisation and the entangled material objects, equipment and spaces through which humans are required to act and interact [82]. These factors have an impact on the allocation and synchronisation of work activities. These findings identify some of the *mechanisms* that trigger different outcomes. Our findings revealed a number of *outcomes* as illustrated by the temporal transformation in the way that blood collection processes within the Central Specimen Reception area were carried out, affecting not only the way that work was undertaken but also how it was sequenced and distributed within a socio-material space that extended from the department across to the whole hospital.

**Table 1:** Case study findings and their connection to components of the EEOC framework

**EEOC framework**	**Impact on laboratory setting**
**Communication**	
How is information exchanged?	Synchronous (e.g., telephone calls) vs. asynchronous (e.g., computer messages) (Blood Bank)
	Paper vs. electronic orders (Haematology and Clinical Chemistry)
Type of information exchange?	Reasons for telephone calls in the Blood Bank (e.g., questions, confirmation, dispensing advice etc) (Blood Bank)
	Decision support systems (e.g. notification of redundant test request) .
How is information processed?	Linear (e.g., order, process and result) aspects vs. collaborative processes (e.g., advice about orders, confirmation of orders and blood products) (Blood Bank)
	Autoverification requirements (Haematology)
What are the outcomes of the information exchange?	Test result reports, blood products (Blood Bank)
	Storage of specimens (e.g., tracking information) (Clinical Chemistry)
	Blood collection requests and patient procedures (Central Specimen Reception)
**Temporal**	
How is communication scheduled?	Accuracy and message reinforcement required for orders (Blood Bank)
How is information synchronised?	Warning and notification systems to ensure notification and synchronisation of work (Blood Bank)
	Synchronisation of systems to check reference ranges, critical values etc. (Haematology)
	Work roles and work process changes (Central Specimen Reception)
How is information allocated?	Sorting and collation of blood collection requests (Central Specimen Reception)
	Frequency and duration of administration tasks (Central Specimen Reception)
How is time conceptualised?	Clock time (e.g. test result turnaround times) (Clinical Chemistry)
	Work flows (e.g. specimen transportation times) (Central Specimen Reception)
**Organisational**	
How is work controlled?	Audit trails (e.g., monitoring of processes) (Blood Bank)
	The role of specimen tracking systems (Clinical Chemistry)
	Changes in work patterns and procedures (Central Specimen Reception)
How is work planned?	Accuracy of orders provided (e.g., establishing the meaning of orders) (Blood Bank)
	Work flow management (Clinical Chemistry)
	Specimen and request collection (Central Specimen Reception)
How is work organised (staffed)?	Accessioning orders, providing test results and dispensation of blood products (Blood Bank)
	Staff availability notifications (Central Specimen Reception)
	Location of activities (e.g. changes to blood collectors work patterns) (Central Specimen Reception)

One of the recognised values of information systems is their ability to integrate departments and organisations [83]. The functioning of new HIT systems is therefore reliant on a number of factors that may not always be evident through the prism of a single part of the pathology service or hospital. Our study of the Blood Bank identified that the use of asynchronous messaging about the ordering, preparation and delivery of blood products depended on communication transactions across the hospital environment to ensure the proper and safe exchange of blood products. Similarly, the transformation of Central Specimen Reception blood collectors’ tasks was triggered by changes in the synchronisation and location of many of their activities across the hospital. The occurrence of change therefore needs to be understood within a temporal context. This requires an assessment of when change takes place, the rate at which it happens and even the extent to which it occurs. Conversely it is also necessary to ascertain what things have remained stable and unchanged [84].

New HIT systems do not work just because they have been built to do so [85]. In some cases change can be attributed to the new technology, in other situations there may be organisational forces that hamper or enhance the potential for change [86]. An EEOC framework seeks to account for the complex range of contextual factors and triggers (mechanisms) that play multiple roles. These triggers are *contingent* and depend on the conditions in which they operate [87]. This is a generative conception of causality, in which causal powers reside not just in the HIT system, but also in the organisational communication relationships and social-material structures of the wider environment [17].

## Conclusions

The adoption and successful implementation of HIT is not simply a matter of matching new technology to organisational need [75] and then proceeding to “roll-out” or “diffuse” the new system [88]. Such approaches ignore the mutual transformation of the organisation by the technology, and of the HIT system by the organisation [88]. This is an important consideration particularly given the dangers associated with the poor planning and implementation of HIT, and the potential for unintended adverse consequences [89,90], workarounds (caused by situations when technology does not fit normal work flows) [38,91], and risks to the quality and safety of patient care [92]. EEOC provides a theoretical lens which can be used to identify and frame important HIT implementation and adoption issues to inform administrators and planners. Healthcare innovation is a collective process which includes a myriad of actors, materials and stakeholders. As such, it is best to view innovation as a product of the complex interactions between the organisation, and key information and communication processes involving the new technology and its users [54]. Communication is a key *constitutive* factor in this process because it is part of a social interaction system directed toward a designated set of outcomes [93]. In this way theoretical representations such as the EEOC framework can be valuable tools to support the resolution of challenges associated with HIT-enabled innovation by providing rich sources of evidence to undertake future research, management planning and policy development.

## Competing interests

The authors declare that they have no competing interests.

## Authors’ contributions

AG, JIW and JB were involved in the design of the study, analysis of results and writing of the paper. AG was primarily responsible in the collection of data. All authors participated in critical revisions of the paper for important intellectual content and its final approval before submission. All authors read and approved the final manuscript.

## Pre-publication history

The pre-publication history for this paper can be accessed here:

http://www.biomedcentral.com/1472-6947/12/68/prepub
